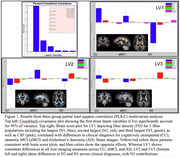# Multivariate analysis of white matter crossing fibers reveals correlations with aMCI and dementia diagnoses

**DOI:** 10.1002/alz.093731

**Published:** 2025-01-09

**Authors:** Andrew R. Bender, Caleb Weissman, Norman Scheel, Youjin Jung, Jessica S. Damoiseaux, Scott J Peltier, Benjamin M. Hampstead

**Affiliations:** ^1^ Michigan Alzheimer’s Disease Research Center, Ann Arbor, MI USA; ^2^ Michigan State University, Grand Rapids, MI USA; ^3^ Michigan State University, East Lansing, MI USA; ^4^ Wayne State University, Detroit, MI USA; ^5^ University of Michigan, Ann Arbor, MI USA

## Abstract

**Background:**

Diffusion magnetic resonance imaging (dMRI) permits characterizing differences in white matter microstructure associated with amnestic mild cognitive impairment (aMCI) and Alzheimer’s dementia (AD). However, most dMRI measures aggregate signals across multiple axonal fiber populations with varying spatial orientations, which limits the sensitivity and specificity of clinical diagnosis. To overcome this shortcoming, we estimated fiber density (FD) measures, independently from crossing fiber populations, and extracellular cerebral spinal fluid (CSF). We hypothesized that aMCI and AD diagnoses are associated with differential patterns of FD changes in larger and smaller diameter fiber populations.

**Method:**

We evaluated cross‐sectional dMRI data from 179 clinically characterized participants enrolled in the University of Michigan Memory and Aging Project. Image processing leveraged the MRtrix3 multi‐shell multi‐tissue fixel‐based analysis framework to estimate FD, separately for three fiber orientations, and CSF. Data analysis used multi‐block partial least squares correlation (PLS‐C) to estimate factors from multidirectional FD and CSF images correlated with differences between cognitively unimpaired (CU; n = 98) and those diagnosed with aMCI (n = 52) or AD (n = 29).

**Result:**

The PLS‐C model yielded three significant latent variables (LVs; Fig. 1), reflecting patterns of both significant positive and negative associations between diagnosis and FD in crossing fibers. LV1 explained 80% of the differences from CU to aMCI to AD, demonstrating a stepwise reduction of FD and increased CSF with greater disease severity. However, LV2 and LV3 showed FD differences in smaller crossing fibers, distinguishing clinical diagnoses. Intriguingly, participants in the aMCI and AD groups showed different regions with increased or decreased FD in smaller crossing fibers, relative to CU participants. Pairwise PLS‐C models showed aMCI and AD diagnoses were associated with similar patterns of FD changes in smaller crossing fibers in overlapping regions.

**Conclusion:**

The present study found distinctive patterns of white matter alterations that systematically differ across diagnostic severity in MCI and Alzheimer’s dementia. These results highlight the value of decomposing signals from crossing fibers as a sensitive neuroimaging correlate to clinical diagnosis. These findings challenge the common perspective that MCI and Alzheimer’s dementia are associated with monotonic declines in white matter integrity.